# Meeting at the Mansion House

**Published:** 1920-10-23

**Authors:** 


### GREAT NORTHERN HOSPITAL.

### Meeting at the Mansion House.

At the large meeting held on St. Luke's Day at the Mansion House, under the chairmanship of the Lord Mayor, in aid of the Great Northern Central Hospital, some interesting figures were disclosed by the Marquess of Northampton, Chairman of the institution. He appealed for funds on the grounds that the Great Northern is run on the most economical lines. On an average the beds of the large London general hospitals cost £155 each per annum to maintain; at the Great Northern the figure is only £143. The institution is unhappily placed in that its revenue from vested capital of endowments is under £1,500 a year. The proportion of nurses is one to every two and three-quarter patients, but though in many hospitals it is far lower—one nurse to three and a-half and even four patients—the work at the Great Northern is particularly arduous, as the 200 beds are so urgently needed—there is a waiting-list of 250—that all but acute cases are as soon as possible drafted on to the convalescent homes. As regards the increase in expenditure, it now costs £5 to open the operating-theatre; the salaries of the nursing staff are figured at £4,500, as against £1,630 in 1913; the rise in the price of the loaf means an increase of £10 weekly; milk now costs £2,600 per annum, as against £840 before the war; and the bill for drugs, instruments, and dressings is £7,500, as against £2,880. To help in some small way towards the high expenditure, contributory systems have been arranged—1s. to 21s. in the general wards per week; one to four guineas in cubicle wards; and four to six guineas in the private rooms. Out-patients pay 4d. a visit.

Before making her speech at the meeting, the Lady Mayoress began by saying "My Lord Mayor," and it was noticed that she turned to her husband and bowed ceremoniously before proceeding. On all sides one hears of the good work done during the last twelve months by the Lady Mayoress. She has almost invariably accompanied the Lord Mayor when he has distributed prizes at the many orphanages, schools, training-ships, etc., during his year of office. Lady Cooper has identified herself very prominently with the work-girls in the City, and she is beloved by her domestic staff at the Mansion House.

Mr. Kennedy Jones, M.P., was at a great disadvantage at the meeting, insomuch as he had to follow immediately after the Bishop of Willesden, who is one of the best platform speakers now living. Mr. Kennedy Jones is a member of the Board of the Hospital, and he appeared to have all the figures connected with it at his fingers' ends. He has a curious habit of frequently moving his right arm when addressing an audience, and it would be difficult to say whether his accent is more Irish than it is Yorkshire. In any case, he impresses everybody by his downright earnestness.

A meeting of the League of the Roses of the Hospital will be held at Islington Town Hall on October 26, at 3 p.m., when the speaker will include Miss Cecily Hamilton and Prebendary C. J. Proctor. The hospital has received £500 from Alexandra Day Fund.

